# GWAS by GBLUP: Single and Multimarker EMMAX and Bayes Factors, with an Example in Detection of a Major Gene for Horse Gait

**DOI:** 10.1534/g3.118.200336

**Published:** 2018-05-10

**Authors:** Andres Legarra, Anne Ricard, Luis Varona

**Affiliations:** * INRA (Institut National de la Recherche Agronomique), UMR 1388 GenPhySE, F-31326 Castanet-Tolosan, France; † INRA (Institut National de la Recherche Agronomique), UMR 1313 GABI, 78352 Jouy-en-Josas, France; ‡ IFCE (Institut Francais du Cheval et de l’Equitation), Recherche et Innovation, 61310 Exmes, France; § Departamento de Anatomía, Embriología y Genética, Universidad de Zaragoza, 50013 Zaragoza, Spain; ** Instituto Agroalimentario de Aragón (IA2), 50013 Zaragoza, Spain

**Keywords:** association analysis, single marker regression, QTL, GWAS, Bayesian regression, GenPred, Shared Data Resources, Genomic Selection

## Abstract

Bayesian models for genomic prediction and association mapping are being increasingly used in genetics analysis of quantitative traits. Given a point estimate of variance components, the popular methods SNP-BLUP and GBLUP result in joint estimates of the effect of all markers on the analyzed trait; single and multiple marker frequentist tests (EMMAX) can be constructed from these estimates. Indeed, BLUP methods can be seen simultaneously as Bayesian or frequentist methods. So far there is no formal method to produce Bayesian statistics from GBLUP. Here we show that the Bayes Factor, a commonly admitted statistical procedure, can be computed as the ratio of two normal densities: the first, of the estimate of the marker effect over its posterior standard deviation; the second of the null hypothesis (a value of 0 over the prior standard deviation). We extend the BF to pool evidence from several markers and of several traits. A real data set that we analyze, with ours and existing methods, analyzes 630 horses genotyped for 41711 polymorphic SNPs for the trait “outcome of the qualification test” (which addresses gait, or ambling, of horses) for which a known major gene exists. In the horse data, single marker EMMAX shows a significant effect at the right place at Bonferroni level. The BF points to the same location although with low numerical values. The strength of evidence combining information from several consecutive markers increases using the BF and decreases using EMMAX, which comes from a fundamental difference in the Bayesian and frequentist schools of hypothesis testing. We conclude that our BF method complements frequentist EMMAX analyses because it provides a better pooling of evidence across markers, although its use for primary detection is unclear due to the lack of defined rejection thresholds.

Bayesian models including simultaneously all marker effects are becoming very popular for GWAS analysis (Habier *et al.* 2011; Wang *et al.* 2012, 2016; Moser *et al.* 2015). The most frequently used prior for marker effects is the normal distribution, known as RRBLUP or SNP-BLUP (Habier *et al.* 2007; VanRaden 2008), which is equivalent to GBLUP (VanRaden 2008), also known in the human literature as GCTA analysis (Yang *et al.* 2010). GBLUP is simple and can be generalized to marker data missing in a large fraction of individuals in the so-called Single Step methods (Aguilar *et al.* 2010; Christensen 2012), and also to multiple traits, or complex models (random regression, genotype by environment, etc.). Because of the equivalence of GBLUP and SNP-BLUP, it is straightforward to obtain from Single Step methods estimates of marker effects for complex traits like, *e.g.*, multiple trait maternal effects (Lourenco *et al.* 2015) or genotype-environment models (Jarquín *et al.* 2014).

Therefore, GWAS can be done exploiting results of GBLUP (Wang *et al.* 2012; Dikmen *et al.* 2013; Casiró *et al.* 2017). Most of these works (*e.g.*, (Wang *et al.* 2012; Dikmen *et al.* 2013) do not report classical statistics neither p-values, whereas standard GWAS by fixed regression “one marker at a time” (*e.g.*, EMMAX (Kennedy *et al.* 1992; Kang *et al.* 2010; Teyssèdre *et al.* 2012)) yields a normal test, *i.e.*, dividing the estimate of the marker effect by its standard error of the estimate, with associated p-values. Remarkably, Gualdrón-Duarte *et al.* (2014) and Bernal-Rubio *et al.* (2016) proved that in (SS)GBLUP or SNP-BLUP, dividing the estimate of the marker effect by its standard error is mathematically equivalent to fixed regression EMMAX, even if markers are estimated as random effects in GBLUP and as fixed effects in EMMAX. In addition, Chen *et al.* (2017) generalized the single marker EMMAX test to a multiple marker test that considers simultaneous sets of markers. In this test, signals from neighboring markers are pooled to create a single p-value measuring strength of association.

This paper has two objectives. The first one is to show that, in addition to previous frequentist tests (single marker and multiple marker EMMAX) it is possible to obtain from GBLUP analysis single marker and multiple marker Bayes Factors (BF) as strength of evidence for the presence or absence of a QTL. In short, the BF is the ratio of probabilities of the data given two competing models (Kass and Raftery 1995) and has been often used in QTL mapping (Heath 1997; Varona *et al.* 2001; Wakefield 2009, 2012; Varona 2010; Habier *et al.* 2011; Legarra *et al.* 2015). The BF empirically seems to provide a consistent procedure across traits and species (Legarra *et al.* 2015). In current Markov Chain MonteCarlo implementations, computation of the BF require indicator variables for “null” or “non null” effects of markers (Habier *et al.* 2011; Legarra *et al.* 2015) and does not include the extensively used GBLUP and SNP-BLUP. In this work, we show how the BF can be easily computed from results of SNP-BLUP or GBLUP, for evidence of a single loci or a set of loci (possibly contiguous). The resulting BF considers correctly both the estimated effect and its incertitude, at one or several loci.

The second objective is to illustrate properties of these two procedures (single and multiple marker EMMAX and BF), plus a Bayesian multiple marker regression (BayesCPi), by analysis of a challenging small horse real data set with presence of a known, yet barely significant, major gene (DMRT3) for gait.

## Material and Methods

### Distributions of marker effect estimates

The methods use the prior (before observing the data) and posterior (estimates and associated errors) distributions of marker effects assuming *a priori* multivariate normality (*i.e.*, SNP-BLUP or GBLUP). Most theory can be found in (Gualdrón Duarte *et al.* 2014; Bernal Rubio *et al.* 2016; Chen *et al.* 2017) and we include it in the Appendix for completion. We will assume throughout that variance components are known; this is a frequent assumption that allows obtaining of closed-form estimators. In particular, variance components can be estimated beforehand (*e.g.*, by REML), or (making strong assumptions) they can be borrowed from pedigree analysis. In either case, a point estimate is used “as if” it was exact, which results in optimistic results. The main notation that we need is a vector of marker effects ***a*** normally distributed with *a priori* mean **0** and variance $I\sigma_{a}^{2}$, and their prediction error variance $C^{aa}$.

### EMMAX tests of association from GBLUP

This section is a reminder of (Gualdrón Duarte *et al.* 2014; Bernal Rubio *et al.* 2016; Chen *et al.* 2017) and we include it here for completeness.

#### Single marker:

The single marker EMMAX procedure is a normal test obtained, in our notation, with the statistic

$$t\;=\;\frac{\hat{a}}{sd\left(\hat{a}\right)}$$

where $\hat{a}$ is the marker estimate of the locus under consideration, obtained from a single SNP-BLUP evaluation (or an equivalent model), and where $sd\left(\hat{a}\right)$ is the frequentist distribution (over conceptual repeated sampling of y) of the SNP-BLUP estimator of the effect a. Somewhat surprisingly, the numerical value of *t* is the same as if *a* was fit as a “fixed regression” GWAS and therefore the distribution of *t* under the null is N(0,1) (Bernal Rubio *et al.* 2016). For instance, assume that $\sigma_{a}^{2}=0.2$ is the *a priori* variance of marker effects. Output of the SNP-BLUP gives an estimate of the marker effect ${\hat{a}}_{i}=0.5$ with a standard deviation of the posterior distribution $s.d.\left(a_{i}|y\right)=0.05$. With these numbers, the frequentist $Var\left({\hat{a}}_{i}\right)=\sigma_{a}^{2}-Var\left(a_{i}|y\right)=0.2-{\left(0.05\right)}^{2}=0.1975$. Thus, $t=\frac{0.5}{0.1975}=2.84$ which yields a p-value of 0.006.

#### Multiple marker:

Consider a subset of *n* markers (possibly consecutive), starting at marker *i*. The statistic is a quadratic form $x={\hat{a}}_{\left[i,i+n\right]}^{\prime }{\left(\Sigma_{\left[i,i+n:i,i+n\right]}\right)}^{-1}\;{\hat{a}}_{\left[i,i+n\right]}$, where $\Sigma =Var\left(\hat{a}\right)=I\sigma_{a}^{2}-C_{\left[i,i+n:i,i+n\right]}^{\mathrm{aa}}$ is the frequentist covariance of these marker effects. Chen *et al.* (2017) proved that under multivariate normality the quadratic form *x* follows a chi-square distribution of *n* degrees of freedom, which yields p-values for the multiple marker EMMAX. Alternatively, derivation of the Hotelling-t squared test, that tests whether a set of correlated sample means are simultaneously different from zero, yields the same result. The previous normal test for the single marker EMMAX is also equivalent to the chi-squared test. Matrix ***Σ*** takes into account uncertainty and collinearity of marker estimates.

For instance, consider two markers with effects $\hat{a}=(0.5,\;0.4)$ (similar effects) with $C^{aa}=\left(\begin{array}{cc} 0.05 & -0.02\\ -0.02 & 0.08 \end{array}\right)$ (estimates of effects are negatively correlated because of linkage disequilibrium) and $\sigma_{a}^{2}=0.2$. The quadratic form has value x=2.61 with p-value 0.27. The evidence given by the p-value lowers because the two effects are correlated.

### Bayes Factors from GBLUP

In this section we include our original derivations.

#### Single marker:

There are two competing models in the BF: that the marker *i* with effect $a_{i}$ “has some effect” (^1^H: $a_{i}\neq 0$) or «has 0 effect» (H0: $a_{i}=0$), and the BF can be written as

$$BF=\frac{p_{H1}\left(y\right)}{p_{H0}\left(y\right)}$$

The BF measures whether the data y is more probable under either of the hypothesis. This can be written, alternatively, as

$$BF=\frac{p\left(y|a_{i}\neq 0\right)}{p\left(y|a_{i}=0\right)}$$ (1)

where $a_{i}$ is the effect of the marker. Typically, this involves a complex MCMC integration. In the particular case of multivariate normality with known variances, Varona *et al.* (2001), Varona (2010) showed that the expression (1) is equal to

$$BF=\frac{p\left(a_{i}=0\right)}{p\left(a_{i}=0|y\right)}$$ (2)

where $p\left(a_{i}=0\right)$ is the density of $a_{i}$ *a priori* evaluated at $a_{i}=0$, and $p\left(a_{i}=0|y\right)$ is the density of $a_{i}$ *a posteriori* evaluated at $a_{i}=0$. Computation of BF using (2) is straightforward because p() is a normal density. In particular, $p\left(a_{i}=0|y\right)$ is the density of $a_{i}=0$ knowing that there is an estimate ${\hat{a}}_{i}$ with a certain *a posteriori* variance $Var\left(a_{i}|y\right)$ (*e.g.*, different for each data set). In algebraic form this is

$$BF=\frac{N\left(0|0,\sigma_{a}^{2}\right)}{N\left(0|{\hat{a}}_{i},Var\left({\hat{a}}_{i}\right)\right)}$$ (3)

where N(x|y,z) is the density of x in the normal distribution with mean y and variance z. Consider the same example as before: $\sigma_{a}^{2}=0.2$, ${\hat{a}}_{i}=0.5$, $s.d.\left(a_{i}|y\right)=0.05$. The BF is thus, in R code:

dnorm(0,0,sqrt(0.2))/dnorm(0,0.5,0.05)

which is 20.76 in the log10 scale. According to Kass & Raftery (1995) this is « Very Strong » evidence.

#### Multiple marker:

Evidence from several consecutive markers in a segment can be pooled together using the BF. Expression (3) is generalized to several SNP markers (markers from *i* to *n*) as:

$$BF=\frac{MVN\left(0|0,I\sigma_{a0}^{2}\right)}{MVN\left(0|{\hat{a}}_{\left[i,i+n\right]},C_{\left[i,i+n:i,i+n\right]}^{\mathrm{aa}}\right)}$$ (4)

where MVN is the density of a multivariate normal distribution and $C_{\left[i,i+n:i,i+n\right]}^{\mathrm{aa}}$ is the posterior (co)variance matrix between the marker estimates. Posterior covariance matrix $C_{\left[i,i+n:i,i+n\right]}^{\mathrm{aa}}$, which is a submatrix of $C^{aa}$, takes into account colinearity between markers caused by LD. In this case, the BF tests whether a set of markers are all simultaneously 0, against the alternative that some of them (if not all) are different from zero.

Consider the same example as before: two markers with effects $\hat{a}=(0.5,\;0.4)$, $C^{aa}=\left(\begin{array}{cc} 0.05 & -0.02\\ -0.02 & 0.08 \end{array}\right)$, $\sigma_{a}^{2}=0.2$. The BF can be computed in R as

dmvnorm(c(0,0),mean = c(0,0),sigma = diag(0.2,2))/dmvnorm(ahat,mean = c(0,0),sigma = Caa) yielding a BF of 1.65 in the log10 scale, lower than the single marker analysis. In a way, this reflects that there is a confusion of marker effects.

#### Multiple trait:

Above methods can be easily extended to the multiple trait case. Multiple trait genomic predictions can be done from Bayesian regressions, SNP-BLUP or (Single Step) GBLUP (Tsuruta *et al.* 2011; Jia and Jannink 2012; Maier *et al.* 2015). Then, the EMMAX tests or the BF for several traits (and possibly markers) simultaneously is very similar to the “Several markers” case considering joint estimates of marker effects $\hat{a}$ for the *n* traits, the *a priori* covariance among marker effects for the *n* traits $K_{0}$, and the *a posteriori* covariance matrix of marker effect estimates $C^{aa}$. Vector ***a*** can include either one or several markers. Typically $K_{0}$ is a function of $G_{0}$, the genetic covariance among traits.

#### Data:

We used a horse real data set to explore and illustrate the properties of the procedures. We also did a limited number of simulations but we chose not to present them as this was extensively done in (Chen *et al.* 2017).

A single base polymorphism at the gene DMRT3 in chromosome 23 has a strong effect on horse ambling gaits (Andersson *et al.* 2012). In French trotters, a SNP marker (marker BIEC2-620109 on chromosome 23 at position 22967656 bp) in strong disequilibrium with this polymorphism has a strong effect in qualification at the race (Ricard 2015; Brard and Ricard 2015). In this work we reanalyzed the same data set, which contains 630 horses and 41711 polymorphic SNP markers. The trait was “outcome of the qualification test”, with a heritability of 0.56. The major gene was not discovered in this data set, and therefore there is no bias due to discovery. We tried the following methods for GWAS:

Bayes factors with the mixture model BayesCPi: (Habier *et al.* 2011) fixing *a priori* that only 0.1% of the markers have an effect (see (Legarra *et al.* 2015) for a full description). This method provides BFs, although our implementation only considers single markers.
Single marker and multiple marker EMMAX tests: as presented in this work, computed via MCMC, up to segments of 100 consecutive markers.
Bayes factors from SNP-BLUP: as presented in this work, computed via MCMC, up to segments of 100 consecutive markers.

EMMAX was fitted using blupf90 (Misztal *et al.* 2002) and homemade scripts, whereas the other used our software GS3 (available at https://github.com/alegarra/gs3), using “OPTION Bayes Factor”. After completion of the analysis, we produced Manhattan plots based on BF and the other statistics; for EMMAX we used Bonferroni corrections to claim genowide significance; for Bayesian procedures, we did not address thresholds for declaring detection; this point will be addressed in the discussion.

### Data availability

The authors state that all data necessary for confirming the conclusions presented in the article are represented fully within the article. Supplemental material available at Figshare: https://doi.org/10.25387/g3.6241928.

## Results

**Figure 1:**
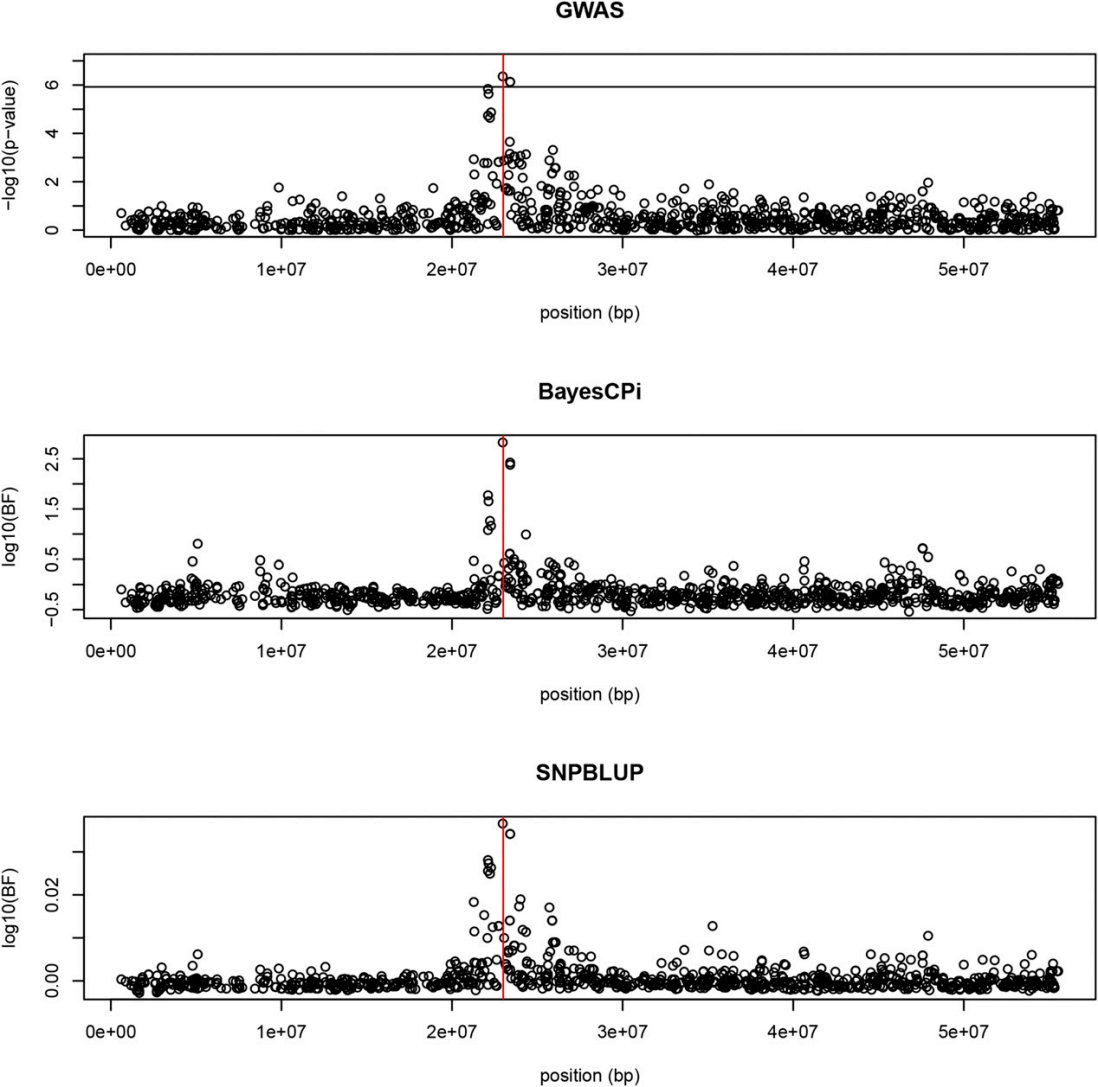
Results (from top to bottom) of single marker regression EMMAX, Bayes Factor for BayesCPi, and Bayes Factor for SNP-BLUP. Bonferroni rejection threshold in EMMAX is 5.9.

**Figure 2:**
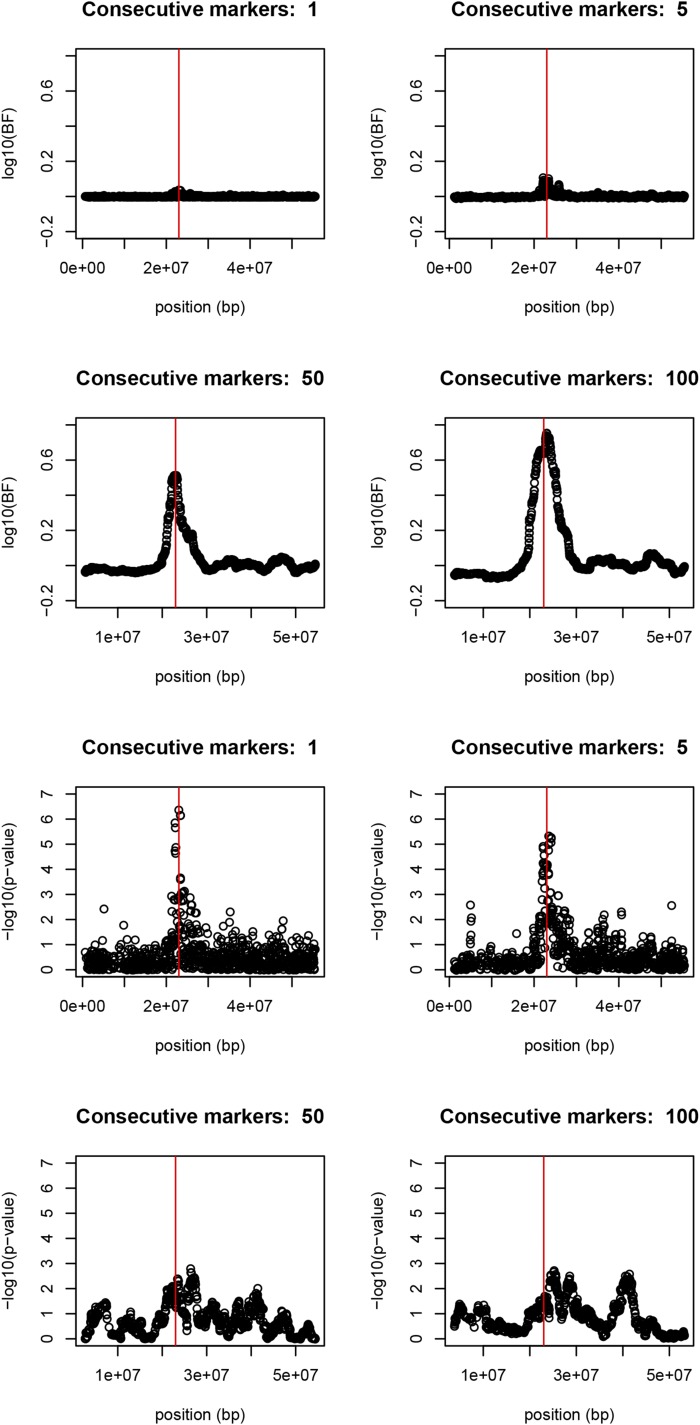
Bayes factor profiles (top) and p-values (bottom) for qualification test in French trotters, chromosome 23. The location of the causal mutation marked with a red vertical bar. Bonferroni rejection threshold is 5.9.

Figure 1 shows results from the single marker association test (EMMAX), the Bayesian multi-marker mixture model GWAS (BayesCPi) and the single marker BF. All three methods point to the SNP (BIEC2-620109 at position 22967656 bp) closest and most associated to the causal gene, and the four significant markers in the EMMAX single marker regression are in LD with each other. This reproduces the results in Ricard (2015). The EMMAX yields significant p-values at the Bonferroni level. Concerning BF, a threshold of 150 (2.17 in the log10 scale) has been suggested (Legarra *et al.* 2015), and the BayesCPi analysis in Figure 2 *does* reach this threshold, but this is not the case in the single marker BF using GBLUP.

In both analyses (BayesCPi and single marker BF), a large number of markers fall below the threshold of 0 in log10(BF), in other words, BF < 1. This means that for those regions the hypothesis that these markers have an effect is *less* likely that the hypothesis that they do *not* have an effect.

Figure 2 shows that evidence of the causal gene increases when using BF across consecutive markers. Systematically, the same location (BIEC2-620109) is spotted. It can be seen that the strength of evidence increase dramatically with increasing consecutive numbers, reaching the suggested “suggestive” threshold of BF > 3 (Kass and Raftery 1995), but not the much higher threshold of 150 suggested (Legarra *et al.* 2015). On the other hand, evidence from EMMAX does actually decrease, becomes non-significant, and, moreover, the highest peak deviates from the true location. This is at first sight a rather surprising result that will be discussed later.

## Discussion

The standard test for GWAS by association analysis is the single marker association analysis (*e.g.*, Kruglyak 1999). Association analysis can account for genetic relationships (Kennedy *et al.* 1992), population structure (Kang *et al.* 2010) and also to a part of individuals not genotyped (Legarra and Vitezica 2015). An alternative is to fit multiple marker simultaneously in the form of Bayesian regression (*e.g.*, Fernando and Garrick 2013). Legarra *et al.* (2015) did not see qualitative differences of Bayesian regressions and association analysis over five data sets and species, and concluded that the interest of Bayesian procedures is to complement regular association analysis. Anyway, Bayesian regression is of interest for three reasons: first, the Bayesian analysis has interesting properties of automatically accounting for multiple test, structure, unbiasedness, false discovery rate and power (Wakefield 2009; Fernando and Garrick 2013); second, genomic evaluation routinely generates marker estimates and these may be used for GWAS; third, complex models used in genetic evaluation can be considered, for instance multiple trait disease all-or-none traits (Parker Gaddis *et al.* 2014).

The analysis that we propose can be seen as an approximation to a mixture analysis such as BayesCPi. For a given marker, we ask the question: “is this marker worth being included in the model?” whereas we pretend that all the other markers are included in the model. Implicitly, the prior is of a normal distribution with a known variance for loci not being tested and a mixture of a point mass at zero and a normal distribution for the locus being tested. In a mixture model (BayesCPi and similar ones), all markers are scrutinized simultaneously, and the strength of evidence compared against the probability value that a marker should be included in the model (usually labeled as *π*). This is probably why the actual numbers for the BF are so different across both methods.

We stress that the SNP-BLUP or GBLUP estimation is run only once, and its results are used to construct BFs for different groups of markers (consecutive or not), if desired. This BF combining information from several markers is quite different from estimating the effect of segments of alleles forming haplotypes, where a haplotype can be seen as a multiallelic marker, and where a different complete estimation must be run for each segment length.

In Bayesian regression models there is a lack of unique criterion to define “relevance” of the association and of corresponding well-defined thresholds; see (Legarra *et al.* 2015) for a description.The numerical values depend strongly on the assumed prior for marker effects (as can be seen in Figure 1). Thus, two researchers fitting, say, BayesCPi and BayesA may obtain different results. The most popular procedure for genomic evaluation and Bayesian regression is SNP-BLUP or its equivalent GBLUP, both of which assume multivariate normality of marker effects. Most often, a reasonable assumption (point estimate) on the variance of marker effects exists, by a transformation of previous estimates of genetic variance (obtained by pedigree analysis or, using the same data set, by genomic REML or similar methods). Using this point estimate underestimates noise linked to estimation of variance components. Here, we present for the first time a closed-form method to estimate BFs for association analysis based on GBLUP results, and we advocate its use. The statistical properties of the BF have been extensively discussed in the statistics literature, but for mapping causal variants it has two very few relevant properties: the BF can show evidence *against* and *for* the null hypothesis, and as data cumulates, the Bayes Factor favors the true hypothesis.

Our results from real data sets show that all methods point to the right marker (the one in stronger LD with the unobserved, but known, QTL). Classical regression analysis is significant and BayesCPi yields a “strong” BF signal. However, the BF observed from SNP-BLUP is 1.07 for the truly associated marker, which is very small support.

Evidence from BF increases when we extend the BF to gather evidence from several markers. A multi – SNP test captures the divergence of the posterior distribution from the 0 vector, and takes into account the posterior dependencies, due to LD, between marker estimates. This is similar to the idea of using the amount of variance explained by each genomic segment (Pérez-Enciso and Varona 2000; Hayes *et al.* 2010; Nagamine *et al.* 2012; Fernando and Garrick 2013). The inconvenience of these methods is mostly computational: they require to do either Restricted Maximum Likelihood (Nagamine *et al.* 2012) or MCMC (Hayes *et al.* 2010; Fernando and Garrick 2013) to estimate variance components, and that only the Restricted Maximum Likelihood estimation has an associated statistical test (Likelihood ratio test), for which consensual threshold exist (such as 0.05 genome-wide corrected by Bonferroni) whereas the MCMC methods use *ad hoc* thresholds that are less consensual. Our proposal does not require MCMC or Restricted Maximum Likelihood, but establishing a threshold for the BF is still ambiguous. An approximate method pools information from estimates of marker effects (Wang *et al.* 2012), but this does not consider not the error in the estimation of marker effects, neither their *a posteriori* correlation in presence of LD. Our proposal is exact, given a point estimate of variance components but does not necessarily require Restricted Maximum Likelihood or MCMC.

The interpretation of the BF in this study is as follows. There are two models, in the first (null) model all markers have 0 effect, whereas in the second (alternative) model at least one of the markers has an effect. In other words, the BF is a contrast between the region contributing, or not, to the genetic variance. When markers’ evidence is pooled across contiguous markers, the evidence for either of the two competing models increases.

Strangely, in our study including more markers in multiple marker EMMAX does not reinforce evidence, contrary to the BF. This is contrary to results of Chen *et al.* (2017). The reason is possibly due to the not-too-strong linkage disequilibrium in our data set, for which p-values do not cumulate information across multiple markers. It would seem that, in our data set, it is more difficult to *disprove* several null hypotheses (null hypothesis in EMMAX: *all* markers are zero) than to *prove* an alternative hypothesis (alternative hypothesis in BF: *some* marker is different from zero).

## Conclusions

We present a Bayesian method (the BF) that complements existing EMMAX methods for QTL detection using marker estimates from SNP-BLUP or (SS)GBLUP from a commonly accepted prior (multivariate normality combined with prior estimates of the genetic variance) and commonly accepted, and used, methods (SNP-BLUP and SSGBLUP). Computations are reasonable and pooling information from several markers is straightforward. Based on our real data set, single marker EMMAX is better to claim significance, whereas multiple marker BF gives a better perspective of influence of LD on the result. This is likely to be data dependent.

## Appendix

### EMMAX tests from GBLUP and SNP-BLUP results

Most of this development is in (Gualdrón Duarte *et al.* 2014; Bernal Rubio *et al.* 2016; Chen *et al.* 2017).

#### SNP-BLUP:

The procedure is easier to be presented from the SNP-BLUP method point of view. In this method, the multivariate prior distribution of marker effect is, for a random locus,

$$p\left(a|\sigma_{a}^{2}\right)=N(0,\sigma_{a}^{2})$$

and $\sigma_{a}^{2}$ is a variance component that usually (but not necessarily) is assumed related to genetic variance in the form $\sigma_{u}^{2}=2\sigma_{a}^{2}\sum^{\text{​}}p_{i}q_{i}$ (Fernando *et al.* 2007; VanRaden 2008). For several loci, $p\left(a|\sigma_{a}^{2}\right)=N(0,I\sigma_{a}^{2})$ *i.e.*, loci effects are assumed uncorrelated *a priori*. A linear model for SNP-BLUP is y=Xb+Za+e where ***Z*** is a matrix of coded genotypes. In SNP-BLUP, the posterior distribution of *a* can be obtained by Markov Chain MonteCarlo (MCMC) (Legarra and Misztal 2008) or from the inverse of the left hand side of Henderson’s Mixed Model Equations:

$$\left(\begin{array}{cc} X^{\prime }X\sigma_{e}^{-2} & X^{\prime }Z\sigma_{e}^{-2}\\ Z^{\prime }X\sigma_{e}^{-2} & Z^{\prime }Z\sigma_{e}^{-2}+I\sigma_{a}^{-2} \end{array}\right)\left(\begin{array}{c} \hat{b}\\ \hat{a} \end{array}\right)=\left(\begin{array}{c} X^{\prime }y\sigma_{e}^{-2}\\ Z^{\prime }y\sigma_{e}^{-2} \end{array}\right)$$ (A1)

In both cases, it is possible to obtain (a) the estimate of the marker effects is $\hat{a}=BLUP\left(a\right)=E(a|y)$, and (b) two measures of incertitude of $\hat{a}$, the (frequentist) sampling variance, *i.e.* $Var(\hat{a})$ and the (Bayesian) posterior variance, *i.e.* Var(a|y). For instance, if the inverse of the right hand side of (A1) is computed:

$${\left(\begin{array}{cc} X^{\prime }X\sigma_{e}^{-2} & X^{\prime }Z\sigma_{e}^{-2}\\ Z^{\prime }X\sigma_{e}^{-2} & Z^{\prime }Z\sigma_{e}^{-2}+I\sigma_{a}^{-2} \end{array}\right)}^{-1}=\left(\begin{array}{cc} C^{bb} & C^{ba}\\ C^{ab} & C^{aa} \end{array}\right)$$

then $Var\left(a|y\right)=C^{aa}$ (Bayesian, conditional on data) and $Var\left(\hat{a}\right)=I\sigma_{a}^{2}-C^{aa}$ (frequentist, over repeated sampling of ***y***). Matrix $C^{aa}$ contains *a posteriori* covariances of marker effects, which reflect allelic frequencies (*i.e.*, a rare SNP is more difficult to estimate) and linkage disequilibrium across markers (two markers in strong LD will have correlated estimates *a posteriori*, and any of them will be less accurate than a marker not in LD with any other). If estimates are obtained by MCMC, $C^{aa}$ can be estimated as the covariance matrix of the samples of the posterior distribution, p(a|y). The R package RRBLUP (Endelman 2011) produces $sd({\hat{a}}_{i})$, and our software GS3 (Legarra *et al.* 2011) can produce parts of $C^{aa}$.

#### GBLUP:

the equivalence between GBLUP and SNP-BLUP implies that marker solutions ($\hat{a}$) can be backsolved for individual solutions ($\hat{u})$ (VanRaden 2008; Strandén and Garrick 2009). Proof is as follows. If individual effects are the sum of marker effects u=Za then ***u*** and ***a*** follow a joint degenerate multivariate normal distribution such that $Var\left(\begin{array}{c} u\\ a \end{array}\right)=\left(\begin{array}{cc} \mathrm{ZDZ'} & \mathrm{ZD}\\ \mathrm{DZ}’ & D \end{array}\right)$. Under the usual assumptions $D=I\sigma_{a}^{2}$ and $\sigma_{u}^{2}=2\sigma_{a}^{2}\sum^{\text{​}}p_{i}q_{i}$ (VanRaden 2008), $\mathrm{ZD}Z^{\prime }=G$ and it can be shown (if ***G*** is invertible) that

$$E\left(\hat{a}|\hat{u}\right)=\frac{1}{2\sum^{\text{​}}p_{i}q_{i}}Z^{\prime }G^{-1}\hat{u}$$

The estimation error of ***a*** from GBLUP estimation is more cumbersome to obtain. Assume that the model for GBLUP is y=Xb+Wu+e, and mixed model equations are:

$$\left(\begin{array}{cc} X^{\prime }X\sigma_{e}^{-2} & X^{\prime }W\sigma_{e}^{-2}\\ W^{\prime }X\sigma_{e}^{-2} & W^{\prime }W\sigma_{e}^{-2}+G\sigma_{u}^{-2} \end{array}\right)\left(\begin{array}{c} \hat{b}\\ \hat{u} \end{array}\right)=\left(\begin{array}{c} X^{\prime }y\sigma_{e}^{-2}\\ W^{\prime }y\sigma_{e}^{-2} \end{array}\right)$$ (A2)

With the inverse of the left hand side equations ${\left(\begin{array}{cc} X^{\prime }X\sigma_{e}^{-2} & X^{\prime }W\sigma_{e}^{-2}\\ W^{\prime }X\sigma_{e}^{-2} & W^{\prime }W\sigma_{e}^{-2}+G\sigma_{u}^{-2} \end{array}\right)}^{-1}=\left(\begin{array}{cc} C^{bb} & C^{bu}\\ C^{ub} & C^{uu} \end{array}\right)$. Gualdrón-Duarte *et al.* (2014) showed that:

$$Var\left(\hat{a}\right)=\frac{1}{2\sum^{\text{​}}p_{i}q_{i}}Z^{\prime }G^{-1}\left(G\sigma_{u}^{2}-C^{uu}\right)G^{-1}Z\frac{1}{2\sum^{\text{​}}p_{i}q_{i}}$$

where $C^{\mathrm{uu}}$ is the element of the inverse of the left hand side matrix corresponding to ***u***; this inverse can be computed by inversion or, again, by MCMC. Finally, the posterior variance of ***a*** is

$$Var\left(a|y\right)=\frac{\sigma_{u}^{2}}{\;2\text{Σ}p_{i}q_{i}}I-\frac{1}{\;2\text{Σ}p_{i}q_{i}}Z^{\prime }G^{-1}(G\sigma_{u}^{2}-C^{uu})G^{-1}Z\frac{1}{\;2\text{Σ}p_{i}q_{i}}$$

This allows computing estimates of marker effects and their errors for very complex models, something difficult to do with standard GWAS or Bayesian Regressions.
